# Supplementary material for: Lipidomic Characterization of Whey Concentrates Rich in Milk Fat Globule Membranes and Extracellular Vesicles
Source: Biomolecules. 2023 Dec 31;14(1):55. doi: 10.3390/biom14010055 (PMC10813332; doi:10.3390/biom14010055)
Supplement: Supplementary file 1 [file biomolecules-14-00055-s001.zip › Figure S1.pdf]

SUPPORTING INFORMATION

Figure S1

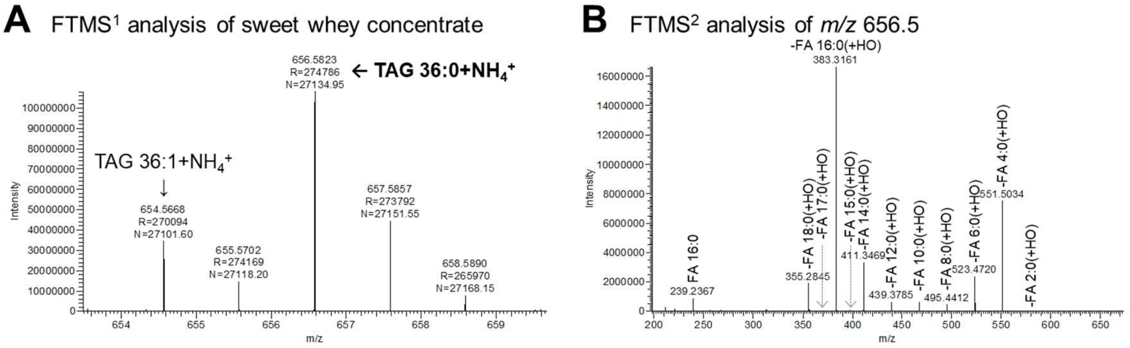

C List of identified molecular lipid species matching TAG 36:0

ID#	Molecular lipid species	ID#	Molecular lipid species	ID#	Molecular lipid species
1	TAG 10:0-10:0-16:0	14	TAG 3:0-5:0-28:0	27	TAG 6:0-15:0-15:0
2	TAG 10:0-11:0-15:0	15	TAG 4:0-12:0-20:0	28	TAG 7:0-11:0-18:0
3	TAG 10:0-12:0-14:0	16	TAG 4:0-14:0-18:0	29	TAG 7:0-12:0-17:0
4	TAG 10:0-13:0-13:0	17	TAG 4:0-15:0-17:0	30	TAG 7:0-13:0-16:0
5	TAG 11:0-11:0-14:0	18	TAG 4:0-16:0-16:0	31	TAG 7:0-14:0-15:0
6	TAG 11:0-12:0-13:0	19	TAG 5:0-11:0-20:0	32	TAG 8:0-10:0-18:0
7	TAG 12:0-12:0-12:0	20	TAG 5:0-13:0-18:0	33	TAG 8:0-11:0-17:0
8	TAG 2:0-14:0-20:0	21	TAG 5:0-14:0-17:0	34	TAG 8:0-12:0-16:0
9	TAG 2:0-16:0-18:0	22	TAG 5:0-15:0-16:0	35	TAG 8:0-13:0-15:0
10	TAG 2:0-6:0-28:0	23	TAG 6:0-10:0-20:0	36	TAG 8:0-14:0-14:0
11	TAG 3:0-13:0-20:0	24	TAG 6:0-12:0-18:0	37	TAG 8:0-8:0-20:0
12	TAG 3:0-15:0-18:0	25	TAG 6:0-13:0-17:0		
13	TAG 3:0-16:0-17:0	26	TAG 6:0-14:0-16:0		

D Quantification of molecular TAG signatures matching TAG 36:0

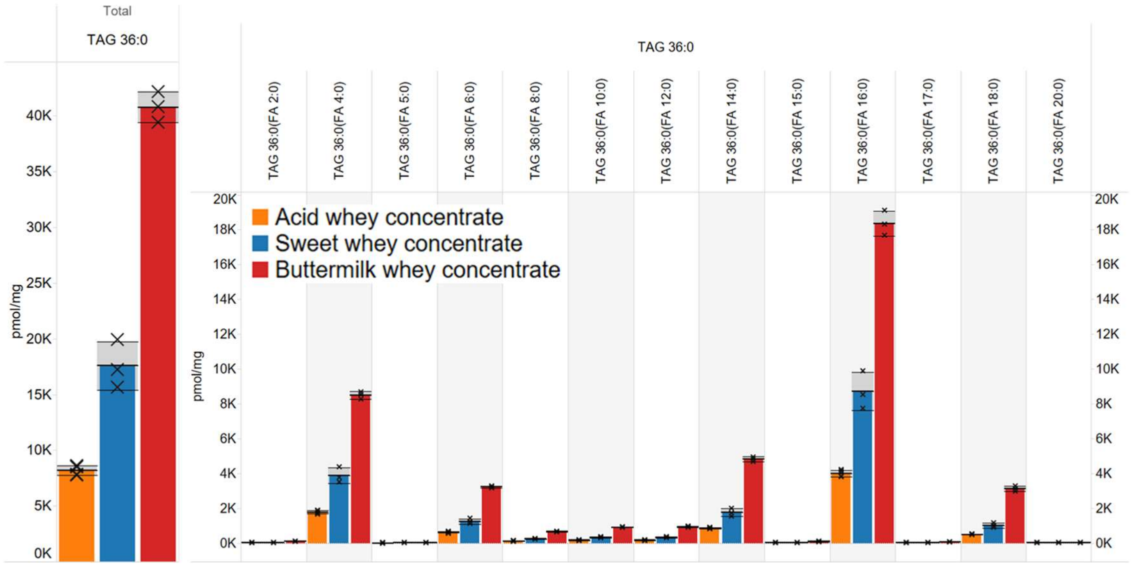

Figure S1. Identification of molecular TAG species and quantification of molecular FA signatures from isomeric TAG molecules. (A) Detection of ammoniated TAG 36:0 in a lipid extract of sweet whey concentrate by high-resolution FTMS1 analysis.(B) Detection of structure-specific fragment ions by high-resolution FTMS2 analysis of the precursor ion at m/z 656.6 (i.e., ammoniated TAG 36:0). Fragments corresponding to individual FA chains are annotated.(C) List of identified, isomeric

molecular lipid species underlying TAG 36:0.(D) Quantification of total TAG 36:0 in the whey concentrates as well as the individual proportions of FA chains making up all isomeric TAG molecules. Note the sum of molar values for all FA chains equals the total amount of TAG 36:0 in each sample.
